# Vorgehen bei Patienten mit Assurity®- und Endurity®-Schrittmachern: Empfehlungen des Nucleus der Arbeitsgruppe Elektrophysiologie und Rhythmologie (AGEP) der Deutschen Gesellschaft für Kardiologie

**DOI:** 10.1007/s00399-022-00906-3

**Published:** 2022-10-25

**Authors:** Carsten W. Israel, Philipp Sommer, Christian Veltmann, Daniel Steven

**Affiliations:** 1 Klinik für Innere Medizin – Kardiologie, Diabetologie & Nephrologie, Evangelisches Klinikum Bethel, Burgsteig 13, 33617 Bielefeld, Deutschland; 2 Klinik Für Elektrophysiologie/Rhythmologie, Herz- und Diabetes-Zentrum Bad Oeynhausen, Bad Oeynhausen, Deutschland; 3 Elektrophysiologie Bremen, Bremen, Deutschland; 4 Klinik III für Innere Medizin, Abteilung für Elektrophysiologie, Herzzentrum, Universitätsklinik Köln, Köln, Deutschland

**Keywords:** Sicherheitsmitteilung, Aggregat-Funktionsstörung, Konnektor, Schrittmacherausfall, Funktionsverlust, Safety advisory, Pacemaker malfunction, Header, Pacemaker recall, Loss of function

## Abstract

Bei einer Untergruppe von Schrittmachern der Modelle Assurity® und Endurity® (Fa. Abbott, Sylmar, Kalifornien, USA; weltweit ca. 83.000 Geräte) kann es bei der Herstellung zu einem Verarbeitungsfehler der Verbindung zwischen Konnektorblock („header“) und Metallgehäuse gekommen sein, wodurch Feuchtigkeit eindringen kann. Dies kann zu Verlust der Telemetriefähigkeit, Umstellen in den Back-up-Modus, verkürzter Batterielaufzeit oder im schlimmsten Fall zu einem Stimulationsverlust führen. Bis Juni 2022 wurden solche Ausfälle bei 128 Geräten weltweit (0,15 %) gemeldet, gesundheitliche Schäden durch einen Ausfall wurden bis zu diesem Zeitpunkt nicht gemeldet.

Der Nucleus der AGEP empfiehlt hierzu folgendes Vorgehen: 1. Patienten, bei denen ein betroffenes Gerät implantiert wurde, sollten informiert werden. 2. Die Risiken für den Patienten im Fall eines Ausfalls des Schrittmachers sollten abgeschätzt werden und die Patienten in „vermutlich schrittmacherabhängig“ (z. B. Indikation: permanenter AV-Block, bei letzter Kontrolle kein Eigenrhythmus, Anteil ventrikulärer Stimulation im Speicher > 90 %), „unklar“ und „vermutlich nicht schrittmacherabhängig“ (z. B. Indikation: Sick-Sinus-Syndrom, bei letzter Kontrolle Eigenrhythmus > 50/min, Anteil ventrikulärer Stimulation im Speicher < 1 %) kategorisiert werden. 3. Bei vermutlich nicht schrittmacherabhängigen Patienten genügt eine Information zusammen mit unveränderter Nachsorge, ggf. können die Nachkontroll-Intervalle auf z. B. 3 Monate verkürzt und/oder eine Fernnachsorge initiiert werden. 4. Bei Patienten mit unklarem Risiko im Fall eines Schrittmacherausfalls sollten zumindest die Nachkontroll-Intervalle auf z. B. 3 Monate verkürzt und/oder ein Fernnachsorge initiiert werden und individuell Nutzen und Risiken eines Geräteaustauschs abgewogen werden. 5. Bei vermutlich schrittmacherabhängigen Patienten wird ein Geräteaustausch empfohlen.

## Abstract (en)

In a subset of patients with pacemaker models Assurity® and Endurity® (Abbott, Sylmar, California, USA; worldwide outside the USA approximately 83,000 devices), a mistake during production may have occurred resulting in insufficient adhesion between the pulse generator header and device housing which in turn may allow moisture to enter the header. This may cause loss of telemetry, switch to back-up mode, reduced battery longevity, or in worst case loss of pacing. Until June 2022, these malfunctions were reported for 128 devices worldwide (0.15%); no permanent harm to patients due to this issue has been reported. The nucleus of the AGEP suggests the following recommendations: (1) Patients with a device under safety advisory should be informed. (2) The risks for the patient in case of loss of stimulation should be assessed. Patients should be categorized into “likely pacemaker-dependent” (e.g., indication permanent atrioventricular [AV] block, no intrinsic rhythm at the last follow-up, percentage of ventricular pacing in the device memory > 90%), “unknown”, or “likely not pacemaker-dependent” (e.g., indication sick sinus syndrome, intrinsic rhythm > 50 bpm at the last follow-up, percentage of ventricular pacing in the device memory < 1%). (3) In likely pacemaker-non-dependent patients, information about this issue should be provided together with an unchanged follow-up or a follow-up with shortened intervals (e.g., every 3 months) and/or remote monitoring. (4) In patients with unknown risk if stimulation failure occurs, at least follow-up intervals should be shortened to, for example, 3 months and/or remote monitoring should be initialized. In addition, risks and benefits of a device exchange should be weighed against each other. (5) In patients who are likely pacemaker-dependent, device exchange is recommended.

## Hintergrund

Die Fa. Abbott hat im Juli 2022 in einer Sicherheitsmitteilung darüber informiert, dass bei einer Untergruppe von ca. 83.000 Herzschrittmachern der Modelle Assurity® und Endurity®, die weltweit außerhalb der USA implantiert wurden (in Deutschland ca. 6500), im Rahmen der Herstellung ein Verarbeitungsfehler bei der Verbindung zwischen Metallgehäuse und Konnektorblock („header“) des Schrittmachers aufgetreten ist. Die potenziell betroffenen Geräte können anhand der Seriennummern identifiziert werden; da der Herstellungsprozess geändert wurde, dürfte dieses Problem bei anderen, neueren Geräten nicht auftreten. Als Folge des Verarbeitungsfehlers kann das Anhaften des Headers an das Metallgehäuse unvollständig sein, und es kann Feuchtigkeit in diese Spalte und von dort aus in den Header selbst gelangen. Dies wiederum kann die Gerätefunktion beeinträchtigen und zum Verlust der Telemetriefähigkeit, automatischem Umstellen in den Back-up-Modus (VVI, 40/min), Verkürzung der Batterielaufzeit und schlimmstenfalls zu einem kompletten Stimulationsverlust führen. Diese Funktionsstörungen wurden der Fa. Abbott bis Juni 2022 in 128 Fällen gemeldet, was einer Häufigkeit von 0,15 % entspricht, und traten im Durchschnitt 2,1 Jahre nach Implantation auf. Eine dauerhafte gesundheitliche Schädigung eines Patienten durch eine dieser Funktionsstörungen wurde der Fa. Abbott bis Juni 2022 nicht gemeldet.

## Konsequenzen

Für das weitere Vorgehen ist eine individuelle Nutzen-Risiko-Abwägung für Patienten mit einem von dieser Sicherheitsmitteilung betroffenen Gerät notwendig. Die berichtete Häufigkeit von Funktionsstörungen dürfte mit 0,15 % deutlich unterschätzt sein, da nicht alle Geräteauffälligkeiten gemeldet werden. Gleiches dürfte für das Risiko einer Patientenschädigung gelten, da Schrittmacher in Deutschland und anderen Ländern postmortal nicht routinemäßig abgefragt werden. Hierbei würde sich ein Verlust der Telemetrie und damit mutmaßlich der Stimulation zeigen. Ein kompletter Stimulationsverlust birgt jedoch ein unterschiedliches Gefahrenpotenzial für Patienten mit und ohne Schrittmacherabhängigkeit. Zudem muss das Risiko eines Stimulationsverlusts gegen das Risiko einer Komplikation bei prophylaktischem Generatorwechsel (Systeminfektion mit Notwendigkeit einer Explantation/Extraktion, Beschädigung der liegenden Schrittmacherelektroden) abgewogen werden. Vor diesem Hintergrund ist ein prophylaktischer Aggregatwechsel bei Patienten, bei denen ein kompletter Funktionsverlust keine unmittelbare Gefahr für die Gesundheit darstellen würde, nicht generell zu empfehlen.

## Beurteilung einer intensivierten Nachkontrolle durch Fernnachsorge („remote monitoring“)

Die Fa. Abbott empfiehlt u. a., bei möglichst vielen Patienten über Merlin.net eine Fernabfrage durchzuführen. Hierbei können engmaschig (z. B. täglich) eine Überwachung der Funktionalität des Geräts durchgeführt und Warnungen einschließlich des *Electronics Performance Indicator* (EPI) erkannt werden. Der EPI erkennt abnormales elektrisches Systemverhalten, das durch einen Verlust der hermetischen Abdichtung verursacht wird. Wenn ein positiver EPI erkannt wird, benachrichtigt Abbott die betreuende Klinik über die E‑Mail-Kontaktinformationen in Merlin.net, die daher aktuell sein müssen. Außer einem positiven EPI würde auch ein „elective replacement indicator“ (ERI) oder ein Verlust der Telemetrie mittels „remote monitoring“ tagesaktuell auf einen (drohenden) Funktionsverlust des Geräts hinweisen.

Einschränkend muss jedoch darauf hingewiesen werden, dass diese Warnungen nur für Patienten sinnvoll erscheinen, die nicht schrittmacherabhängig sind, da die Überwachung nicht kontinuierlich, sondern nur z. B. einmal pro 24 h erfolgt. Problematisch ist weiterhin, dass eine Funktionsstörung inkl. vollständigem Funktionsverlust auch innerhalb einer Woche nach unauffälliger Fernabfrage in Merlin.net und auch ohne Vorboten auftreten kann. Weiterhin ist bei einigen Geräten eine aktive Abfrage durch den Patienten erforderlich (Telemetriekopf über Schrittmacher halten und mittels Telefon übermitteln), was von vielen Patienten nicht durchführbar ist. Schließlich muss davon ausgegangen werden, dass kurzfristig nur eine begrenzte Zahl von Remote-Monitoring-Geräten zur Verfügung gestellt werden kann.

## Empfohlenes Patientenmanagement

Patienten, bei denen eines der unter die Sicherheitsmitteilung fallenden Geräte implantiert wurde, sollten zunächst über die Problematik informiert (z. B. über einen Brief, eine E‑Mail, bei einem kurzfristigen Kontrolltermin oder via Telefon) und diese Informationsweitergabe dokumentiert werden.

Für jeden Patienten sollte das individuelle Risiko ermittelt werden (Tab. 1), das ein Funktionsausfall des Schrittmachers mit sich bringen würde, und er sollte einer der 3 folgenden Kategorien zugeordnet werden:

1. Patienten, die *vermutlich nicht schrittmacherabhängig* sind, wovon z. B. bei der Indikation Sick-Sinus-Syndrom, bei Eigenrhythmus > 50/min während der letzten Kontrolle oder bei einem Anteil ventrikulärer Stimulation im Speicher < 1 % auszugehen ist,
2. Patienten, die *vermutlich schrittmacherabhängig* sind, was sich z. B. ergibt aus der Indikation eines (permanenten) AV-Blocks, dem Befund, dass bei der letzten Kontrolle kein Eigenrhythmus bestand oder einem Anteil ventrikulärer Stimulation im Speicher > 90 %.
3. Alle anderen Patienten sollten als *Schrittmacherabhängigkeit unklar* kategorisiert werden.

### Zu 1.

Bei *vermutlich nicht schrittmacherabhängigen* Patienten sollte eine Information zusammen mit einer unveränderten Nachsorge erfolgen. Gegebenenfalls können die Nachkontroll-Intervalle auf z. B. 3 Monate verkürzt und/oder eine Fernnachsorge initiiert werden. Ein prophylaktischer Schrittmacheraustausch wird nicht empfohlen, einerseits aufgrund der niedrigen Inzidenz relevanter Funktionsstörungen (aktuell 0,15 %), der offenbar sehr niedrigen Inzidenz eines gesundheitlichen Schadens durch einen Funktionsverlust (gemeldet 0 %) und andererseits aufgrund des nicht unerheblichen Risikos eines Gesundheitsschadens durch eine Austauschoperation (Systeminfektion, Beschädigung der liegenden Schrittmacherelektroden), das im Bereich von ca. 0,5–1,0 % anzunehmen ist.

### Zu 2.

Bei *vermutlich schrittmacherabhängigen* Patienten wird ein elektiver Aggregatwechsel empfohlen.

### Zu 3.

Patienten mit *unklarem* Risiko, was im Fall eines Schrittmacherausfalls geschehen würde (z. B. intermittierender AV-Block, ventrikuläre Stimulationshäufigkeit 1–90 %), sollten über Nutzen und Risiko sowohl einer weiteren Verwendung des Aggregats mit Sicherheitsmitteilung als auch eines prophylaktischen Aggregatsaustauschs aufgeklärt werden. In jedem Fall sollte eine Verkürzung der Nachkontroll-Intervalle auf z. B. 3 Monate und/oder eine Fernnachsorge initiiert werden. Die Entscheidung für oder gegen einen prophylaktischen Aggregataustausch sollte individuell (Einschätzung des Arztes, Patientenwunsch nach adäquater Information) und auf Basis verschiedener Überlegungen erfolgen:

- Wie hoch ist das Risiko für eine Asystolie im Fall eines Funktionsverlusts des Schrittmachers (AV-Überleitung vor Schrittmacher, intrinsischer Rhythmus bei der letzten Kontrolle bzw. aktuell, Prozentsatz der ventrikulären Stimulation im Schrittmacherspeicher)? Gegebenenfalls hierzu einmal über eine temporäre Programmierung auf VVI, 30/min den intrinsischen (Ersatz‑)Rhythmus testen.
- Wie hoch ist das Sturzrisiko bei plötzlicher, kürzerer Asystolie (Frailty, Osteoporose, Fähigkeit zu einer raschen Reaktion, die z. B. bei Patienten mit M. Parkinson stark verzögert ist)?
- Wie gut kann der Patient einen Funktionsverlust seines Geräts bemerken und kommunizieren (schlecht z. B. bei Demenz)?
- Könnte der Patient eine tägliche (aktive) Fernüberwachung seines Geräts bewerkstelligen?
- Wäre der Patient zu einer Nachkontrolle in kürzeren Intervallen (z. B. alle 3 Monate) in der Lage?

**Table 1:** 

Patientengruppe	Kriterien	Empfehlung
Nicht SM-abhängig	Indikation Sick-Sinus-Syndrom Bei Kontrolle intrinsischer Rhythmus RV-Stimulation < 1 %	Kein prophylaktischer Austausch Kürzere Kontroll-Intervalle und/oder Remote Monitoring
SM-abhängig	Indikation (permanenter) AV-Block ≥ II° Bei Kontrolle kein intrinsischer Rhythmus RV-Stimulation > 90 %	Prophylaktischer Austausch
Unklar	Indikation z. B. intermittierender AV-Block RV-Stimulation > 1 bis < 90 %	Kürzere Kontroll-Intervalle und/oder Remote Monitoring *Individuell, abhängig von:* a. Patientenwunsch b. Einschätzung des Arztes c. Risiko für Asystolie vs. Ersatzrhythmus (temporäre Testung VVI, 30/min) d. Sturz‑/Verletzungsrisiko e. Fähigkeit des Patienten zur aktiven täglichen Fernabfrage f. Wahrnehmungs‑/Kommunikationsfähigkeit von Funktionsstörungen durch den Patienten

*AV* atrioventrikulär, *RV* rechtsventrikulär, *SM* Schrittmacher

## Zusätzliche Hinweise

Sollte sich bei einer In-office-Nachkontrolle oder Fernabfrage eines der unter die Sicherheitsmitteilung fallenden Geräte eine EPI-Benachrichtigung oder ERI zeigen, sollte das Aggregat unverzüglich ausgetauscht werden. Gleiches gilt selbstverständlich für Geräte mit vollständigem Funktionsverlust, Verlust der Telemetriefähigkeit oder automatischem Rückfall in einen Back-up-Modus.

Bei Patienten, die sich für eine tägliche Fernüberwachung mittels Merlin.net entscheiden, sollte die Fähigkeit, die Abfrage selbst durchzuführen, überprüft werden. Auf die Bedeutung einer täglichen Datenübertragung sollte dezidiert hingewiesen werden.

Die Hinweise und Empfehlungen der Fa. Abbott können unter [1] nachgelesen werden. Unter https://www.cardiovascular.abbott/int/en/hcp/product-advisories/laser-adhesion-safety-lookup.html können Einzelheiten zu den Aggregaten und Fernabfragemöglichkeiten nachgeschlagen werden. Die Fa. Abbott hat das BfArM informiert und sammelt weiter Meldungen zu Problemen und Patientenschädigungen im Rahmen dieser Sicherheitsmitteilung und wird die Informationen ggf. aktualisieren. Daher sollten beobachtete Funktionsstörungen unbedingt an die Fa. Abbott und das BfArM gemeldet werden. Alle explantierten Geräte sollten zur Analyse an die Fa. Abbott geschickt werden. Diese Informationen sollten auch an möglichst alle Mitarbeiter und ggf. Zuweiser, die Schrittmacher kontrollieren, die unter diese Sicherheitsmitteilung fallen, weitergeleitet werden.
